# Combination of anti-CD20 and hetrombopag in relapsed/refractory immune thrombocytopenia: a case series

**DOI:** 10.3389/fmed.2025.1701819

**Published:** 2025-11-21

**Authors:** Xiaolei Zhang, Yang Li, Yujie Guo, Xuquan Zhou, Fengru Lin, Yan Wang, Lina Xing

**Affiliations:** Hebei Key Laboratory of Hematology, Department of Hematology, The Second Hospital of Hebei Medical University, Shijiazhuang, China

**Keywords:** immune thrombocytopenia, anti-CD20 monoclonal antibody, hetrombopag, relapsed/refractory, case series

## Abstract

**Purpose:**

This study aimed to assess the effectiveness of anti-CD20 monoclonal antibody and hetrombopag for relapsed/refractory immune thrombocytopenia (ITP) following glucocorticoid treatment.

**Methods:**

We retrospectively included four patients with relapsed/refractory ITP. The median disease duration is 9 months. Their prior lines of therapy numbered 4, 2, 4, and 2, respectively. They were treated with a combination of anti-CD20 monoclonal antibody and hetrombopag, followed by maintenance therapy with hetrombopag and monitoring of platelet (PLT) changes.

**Results:**

All four patients achieved complete response (CR), with the time to response ranging from 2 to 9 days and the duration of response (DoR) ranging from 3 to 27 months. CR was defined as a PLT count >100 × 10^9^/L and the absence of bleeding. The treatment was well tolerated. Only the first patient’s PLT count decreased to 31 × 10^9^/L following discontinuation of therapy after 6 months of DoR. Accordingly, the patient was treated with avatrombopag monotherapy, which maintained the PLT count at normal levels. The third patient presented with secondary ITP. After treatment with ripertamab-hetrombopag, the patient received combination therapy with hetrombopag, glucocorticoids, tacrolimus, and hydroxychloroquine, with the PLT count being maintained within the normal range. The other two patients remained in sustained CR throughout the follow-up period.

**Conclusion:**

Combining anti-CD20 monoclonal antibody with hetrombopag may offer therapeutic benefits in patients with relapsed/refractory ITP.

## 1 Introduction

Immune thrombocytopenia (ITP) is an acquired autoimmune bleeding disorder characterized by excessive platelet (PLT) destruction and suppressed PLT production mediated by humoral and cellular immunity (1–3). The reported prevalence of ITP is 2/100,000, with secondary ITP accounting for 20% of overall ITP cases (4). Primary ITP is defined as a PLT count of <100 × 10^9^/L without other causes of thrombocytopenia, while secondary ITP is caused by other diseases (e.g., infections, neoplasms, medications, and rheumatic and immune diseases) (5).

The primary goal of ITP treatment is not to normalize the PLT count; instead, it is to maintain it at a level that can prevent the risk of major bleeding. The first-line treatment of ITP often involves intravenous immunoglobulin (IVIG) and glucocorticoids, with IVIG being more commonly used as a salvage treatment (6). Glucocorticoids have several advantages over IVIG, including high effectiveness, wide availability, and low cost. However, long-term glucocorticoid treatment causes adverse effects in ≥20% of patients; furthermore, 70–80% of patients relapse during drug tapering (7). Accordingly, there has been consensus regarding the ineffectiveness of glucocorticoid therapy for ITP (8, 9).

The second-line treatment of ITP primarily involves splenectomy, anti-CD20 monoclonal antibody therapy, or thrombopoietin receptor agonist (TPO-RA). Although splenectomy is an effective treatment, it involves a high risk of surgical complications (10). Anti-CD20 monoclonal antibodies, including rituximab and ripertamab, can bind to B cells, induce Fc receptor-mediated cytolysis, remove excess B cells from the blood, reduce autoantibody production, and prevent excessive PLT destruction (11). The initial response rate to rituximab is as high as 60% (9, 12); however, the durable response rates are 40% at 6–12 months and 20–30% at 5 years (13–15). Further, rituximab has a slow onset of action (4–8 weeks after the initial dose) and an increased bleeding risk before the onset of action (16). Additionally, its strong immunosuppressive effect increases the incidence of infections (17); furthermore, the anti-CD20 monoclonal antibody is expensive, which limits its widespread use in clinical settings. TPO-RA belongs to a class of synthetic TPO analogs that includes eltrombopag, hetrombopag, avatrombopag, lusutrombopag, and romiplostim. Generally, the onset of action of TPO-RA is within 2 weeks. TPO-RA has been shown to be effective in treating 70–80% of patients with refractory ITP, with this effect being observed long term after drug discontinuation in 10–30% of patients. Additionally, TPO-RA has an acceptable safety profile (18–20).

Notably, some patients with ITP who do not show a stable response to any of the available monotherapies may benefit from the synergistic effects of combination therapies. The combination of anti-CD20 monoclonal antibody and TPO-RA could be an ideal choice for the treatment of relapsed/refractory ITP patients. On the one hand, B-cell depletion reduces antibody production; on the other hand, TPO-RA promotes platelet production. However, there are still few studies on the combination of these two drugs for the treatment of ITP worldwide. Accordingly, this study aimed to assess the effectiveness of combination therapy with anti-CD20 monoclonal antibody and hetrombopag for relapsed/refractory ITP.

## 2 Materials and methods

We retrospectively included four patients with relapsed/refractory ITP who were treated with anti-CD20 monoclonal antibody and hetrombopag. Relapse is defined as a recurrence of thrombocytopenia with PLT counts falling below 30 × 10^9^/L or the occurrence of bleeding after remission (8). Refractory is defined as a condition in which PLT counts do not respond to ≥2 treatments, there is no single medication to which they respond, and their platelet counts are very low and accompanied by bleeding (21). The inclusion criteria were isolated thrombocytopenia, age ≥18 years, and PLT count <30 × 10^9^/L. In all patients, a bone marrow smear showed normal leukocytes and erythrocytes, normal or increased megakaryocyte counts, and normal splenic size. We excluded patients with severe cardiac, renal, hepatic, or pulmonary dysfunction; severe immunodeficiency, myelofibrosis, pregnancy, or lactation. All four patients signed informed consent forms. This study was approved by the Ethics Committee of the Second Hospital of Hebei Medical University (approval no. 2025-P032).

PLT counts should be monitored weekly during combination therapy of anti-CD20 monoclonal antibody and hetrombopag. After discontinuing combination therapy, PLT counts should be monitored every 2 weeks. Subsequently, monitoring intervals may be extended to monthly or longer based on PLT levels and medication status. The response rate to combination therapy was assessed as complete response (CR), response (R), or no response (NR). CR was defined as a PLT count >100 × 10^9^/L and the absence of bleeding. R was defined as a PLT count >30 × 10^9^/L, with at least a 2-fold increase in the baseline PLT count, and the absence of bleeding. NR was defined as a PLT count <30 × 10^9^/L or a 2-fold increase in the baseline PLT count or the presence of bleeding. The time to response (TTR) is defined as the time from treatment onset to response. The duration of response (DoR) was defined as the time from response to a PLT count <30 × 10^9^/L (Table 1).

**Table 1:** Clinical features of the study patients.

Clinical features	Case 1	Case 2	Case 3	Case 4
Sex	Male	Female	Female	Male
Age before treatment line (year)	50	55	49	58
Age at ITP diagnosis (year)	49	55	44	58
ITP duration (month)	16	2	60	2
ITP before the treatment line	Chronic	Acute	Chronic	Acute
PLT (×10^9^/L)				
Before the treatment	8	8	16	28
After the treatment	108	655	117	146
Highest level	500	833	235	146
Prior therapy lines	4	2	4	2
Prior treatment	Steroids; rhTPO; TPO-RA; TCM; IVIG	Steroids; TPO-RA; Sirolimus; rhTPO	rhTPO; Steroids; IVIG; Tacrolimus; Hydroxychloroquine; TCM; TPO-RA; Danazol	Steroids; rhTPO; TPO-RA
Study treatment line	RTX + Hetrombopag	RTX + Hetrombopag	Ripertamab+Hetrombopag	Ripertamab+Hetrombopag
Concomitant medications	Danazol	/	GC + TAC + HCQ	/
Maintenance medications	Hetrombopag +Danazol	/	GC + TAC + HCQ	Hetrombopag
Response	CR	CR	CR	CR
TTR (day)	2	9	9	2
Time to CR (day)	44	9	9	2
DoR (month)	23+	27+	29+	12

ITP, immune thrombocytopenia; PLT, platelet; rhTPO, recombinant human thrombopoietin; TCM, traditional Chinese medicine; TPO-RA, thrombopoietin receptor agonist; IVIG, intravenous immunoglobulins; RTX, rituximab; CR, complete response.

### 2.1 Medical history

1) Case 1 involved a 50-year-old man diagnosed with chronic ITP. He had a medical history spanning 1 year and 4 months. Initially, he underwent glucocorticoid treatment. However, his chest computed tomography (CT) indicates old lesions, and a tuberculosis-specific test could not rule out tuberculosis, so glucocorticoids were discontinued. Subsequently, the patient was started on recombinant human thrombopoietin (rhTPO) and eltrombopag, which increased the PLT count to 160 × 10^9^/L. However, the PLT count decreased to <10 × 10^9^/L after eltrombopag discontinuation. Accordingly, he underwent PLT transfusion and received oral eltrombopag, which increased the PLT count to 66 × 10^9^/L. Subsequently, he was started on traditional Chinese herbal medicine, with the PLT count decreasing to ≤10 × 10^9^/L. A repeat bone marrow smear revealed obstruction of megakaryocyte maturation; further, the chromosomal karyotype was 46, XY. Accordingly, he was diagnosed with relapsed persistent ITP and was started on IVIG (0.4 g/kg for 5 days); however, there was no improvement in the PLT count. After ruling out tuberculosis, the patient was started on glucocorticoids (dexamethasone [30 mg, days 1–4], followed by methylprednisolone [1 mg/kg·day] and gradually reduce the dosage, which increased the PLT count to >100 × 10^9^/L. However, after reducing the dosage of methylprednisolone tablets to 32 mg/day, the PLT count rapidly decreased to 11 × 10^9^/L. Therefore, hetrombopag was started at 7.5 mg/day with tapering of the methylprednisolone dosage, which normalized the PLT count. The PLT count fluctuated at the lower limit of normal during the process of hetrombopag tapering. During this time, the patient developed gout; accordingly, he was treated with diclofenac sodium enteric-coated tablets, which sharply decreased the PLT count to 20 × 10^9^/L. Therefore, the hetrombopag dose was re-increased to 7.5 mg/day, which was combined with danazol at 0.2 g twice a day. After 1 month of treatment, there was no significant increase in the PLT count.
2) Case 2 involved a 55-year-old woman diagnosed with acute ITP. She had a 2-month medical history. Initially, her PLT count was 2 × 10^9^/L; furthermore, a bone marrow smear revealed impaired maturation of megakaryocytes, which harbored the TET2 mutation with an allele frequency of 1.00%. The chromosomal karyotype of the bone marrow was 46, XX, with no significant abnormalities in flow cytometry or bone marrow pathology. The patient was successively treated with 15 mg dexamethasone tablets combined with eltrombopag at 50 mg/day for 5 days, 40 mg dexamethasone tablets combined with hetrombopag at 2.5 mg/day for 4 days, hetrombopag at 7.5 mg/day combined with sirolimus 0.5 mg/day, and hetrombopag 7.5 mg/day combined with rhTPO for 14 days. None of these treatments significantly increased the PLT count.
3) Case 3 involved a 49-year-old woman diagnosed with chronic ITP. She had a 5-year medical history. Initially, her PLT count was 12 × 10^9^/L, which normalized after combination therapy with rhTPO and glucocorticoids but subsequently decreased after glucocorticoid reduction. Next, she showed intermittent episodes of skin ecchymosis and thrombocytopenia with glucocorticoid therapy. In 2021, the patient presented to the hospital with thrombocytopenia and was diagnosed with primary Sjögren syndrome. The PLT count normalized after treatment with IVIG and glucocorticoids. Maintenance therapy was administered using methylprednisolone tablets, tacrolimus, and hydroxychloroquine. The PLT count remained normal for approximately 1 year before it decreased again. Subsequently, the patient was intermittently treated with traditional Chinese medicine, eltrombopag, and danazol orally, which normalized the PLT count but did not maintain it. When the PLT count dropped to 1 × 10^9^/L, the patient was treated with rhTPO combined with 25 g IVIG for 5 days, as well as 40 mg methylprednisolone sodium succinate for 4 days. However, this did not significantly increase the PLT count. At this time, a bone marrow smear still showed impaired megakaryocyte maturation accompanied by mild iron deficiency. Fluorescence *in situ* hybridization and bone marrow pathology did not reveal significant abnormalities.
4) Case 4 involved a 58-year-old man diagnosed with acute ITP. He had a 2-month medical history. A bone marrow smear showed impaired megakaryocyte maturation with karyotypes 46 and XY; furthermore, bone marrow pathology revealed a few hematopoietic cells with granulocytes, erythrocytes, and megakaryocytes visible in all three hematopoietic cell lines. Initially, he was treated with 40 mg dexamethasone combined with rhTPO for 4 days, which increased the PLT count to 82 × 10^9^/L. However, the PLT count decreased to 4 × 10^9^/L after the treatment was stopped. It increased to 141 × 10^9^/L after the previous treatment protocol was repeated, followed by hetrombopag 2.5 mg/day as maintenance treatment. After 1 week, the platelet count decreased to 65 × 10^9^/L, and the hetrombopag dosage was increased to 5 mg/day. However, the PLT count decreased to 29 × 10^9^/L, with detectable skin petechiae after 1 week.

### 2.2 Treatment

Anti-CD20 monoclonal antibody was administered at 375 mg/m^2^ for the first week, followed by 100 mg/week for the next 3 weeks. The first patient was treated with rituximab, while the other patients were treated with ripertamab. Initially, hetrombopag was administered orally at 7.5 mg/day in cases 1–3 and at 5 mg/day in case 4; subsequently, it was administered as maintenance therapy after the combination regimen. Prior to initiating combination therapy, case 1 had already been receiving treatment with danazol capsules, which continued throughout the subsequent course of treatment. In case 2, the PLT count reached 393 × 10^9^/L after the second treatment with anti-CD20 monoclonal antibody; accordingly, hetrombopag was discontinued. Since case 3 involved secondary primary Sjögren’s syndrome, glucocorticoids, tacrolimus, and hydroxychloroquine were simultaneously administered. PLT changes were dynamically monitored, with adjustment of the hetrombopag dose based on the PLT count.

## 3 Results

### 3.1 Treatment response

1) Case 1: After receiving rituximab-hetrombopag-danazol therapy, the PLT count increased from 8 × 10^9^/L to 37 × 10^9^/L, with a TTR of 2 days; furthermore, CR was achieved in 44 days. During the period of hetrombopag and danazol, the PLT count was maintained at 61–143 × 10^9^/L for 6 months. However, it decreased to 31 × 10^9^/L at 20 days after hetrombopag discontinuation. The DoR was 23 months; furthermore, the patient did not experience any significant adverse reactions during combination therapy. Subsequently, avatrombopag monotherapy was initiated, which maintained the PLT count at >100 × 10^9^/L, with a peak PLT count of 500 × 10^9^/L.
2) Case 2: After receiving rituximab-hetrombopag therapy for 9 days, the patient achieved CR, with the PLT count increasing from 8 × 10^9^/L to 258 × 10^9^/L. Similarly, the TTR was 9 days. After the second administration of anti-CD20 monoclonal antibody therapy, the PLT count reached 393 × 10^9^/L; therefore, hetrombopag was discontinued. During the follow-up period until July 2025, which represents 27 months after the combination therapy, the patient did not receive any medication for increasing the PLT count, which was maintained at 260–270 × 10^9^/L. The DoR was >27 months; furthermore, the patient did not experience any significant adverse reactions during the combination therapy.
3) Case 3: After receiving rituximab-hetrombopag therapy for 9 days, the patient achieved CR, with the PLT count increasing from 16 × 10^9^/L to 110 × 10^9^/L. Similarly, the TTR was 9 days. After ripertamab therapy, the patient was treated with a combination of hetrombopag, methylprednisolone tablets, tacrolimus, and hydroxychloroquine. After 4 months, hetrombopag was discontinued, with the other three drugs being continued. After the completion of ripertamab-hetrombopag therapy, the PLT count remained within the normal range, with a DoR of >29 months. There were no adverse reactions related to ripertamab and hetrombopag combination therapy.
4) Case 4: After receiving rituximab-hetrombopag therapy for 2 days, the patient achieved CR, with the PLT count increasing from 28 × 10^9^/L to 115 × 10^9^/L. Similarly, the TTR was 2 days. After treatment with ripertamab, the PLT count was 146 × 10^9^/L; accordingly, the hetrombopag dose was reduced to 2.5 mg once daily. The PLT count remained normal, with a DoR of >12 months. There were no adverse reactions related to the combination therapy.

**Figure 1:**
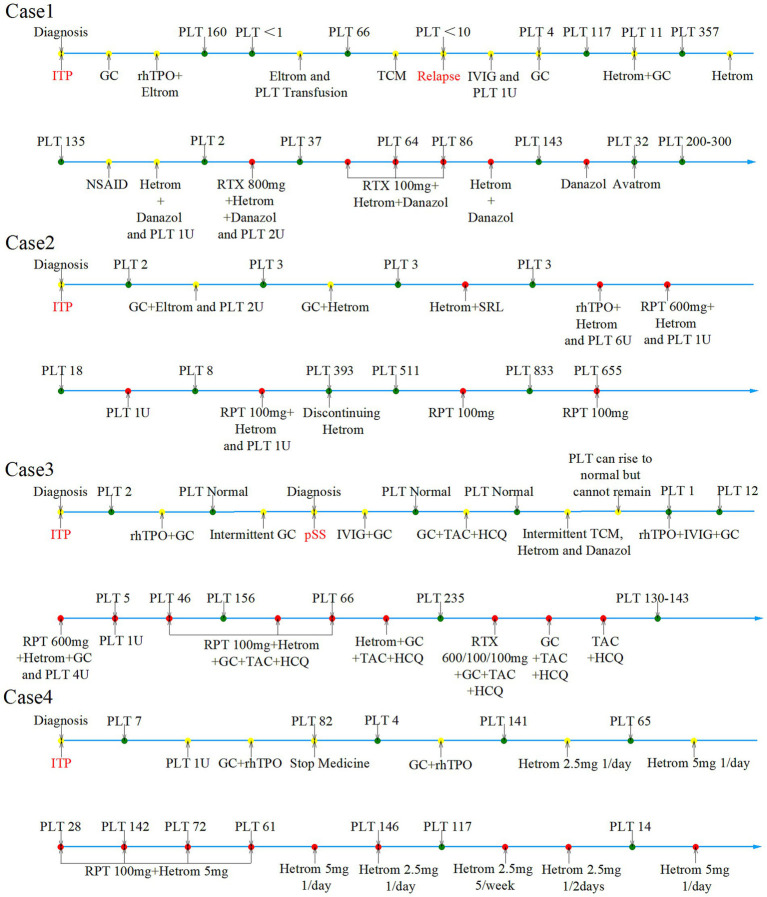
Clinical course of the patients. ITP, immune thrombocytopenia; GC, glucocorticoid; rh TPO, recombinant human thrombopoietin; PLT, platelet (the unit for PLT is ×10^9^/L); Eltrom, eltrombopag; TCM, traditional Chinese medicine; IVIG, intravenous immunoglobulins; U, unit; Hetrom, Hetrombopag; Avatrom, Avatrombopag; NSAIDs, non-steroidal anti-inflammatory drugs; RTX, rituximab; SRL, sirolimus; RPT, ripertamab; pSS, primary Sjögren’s syndrome; TAC, tacrolimus; HCQ, hydroxychloroquine.

**Figure 2:**
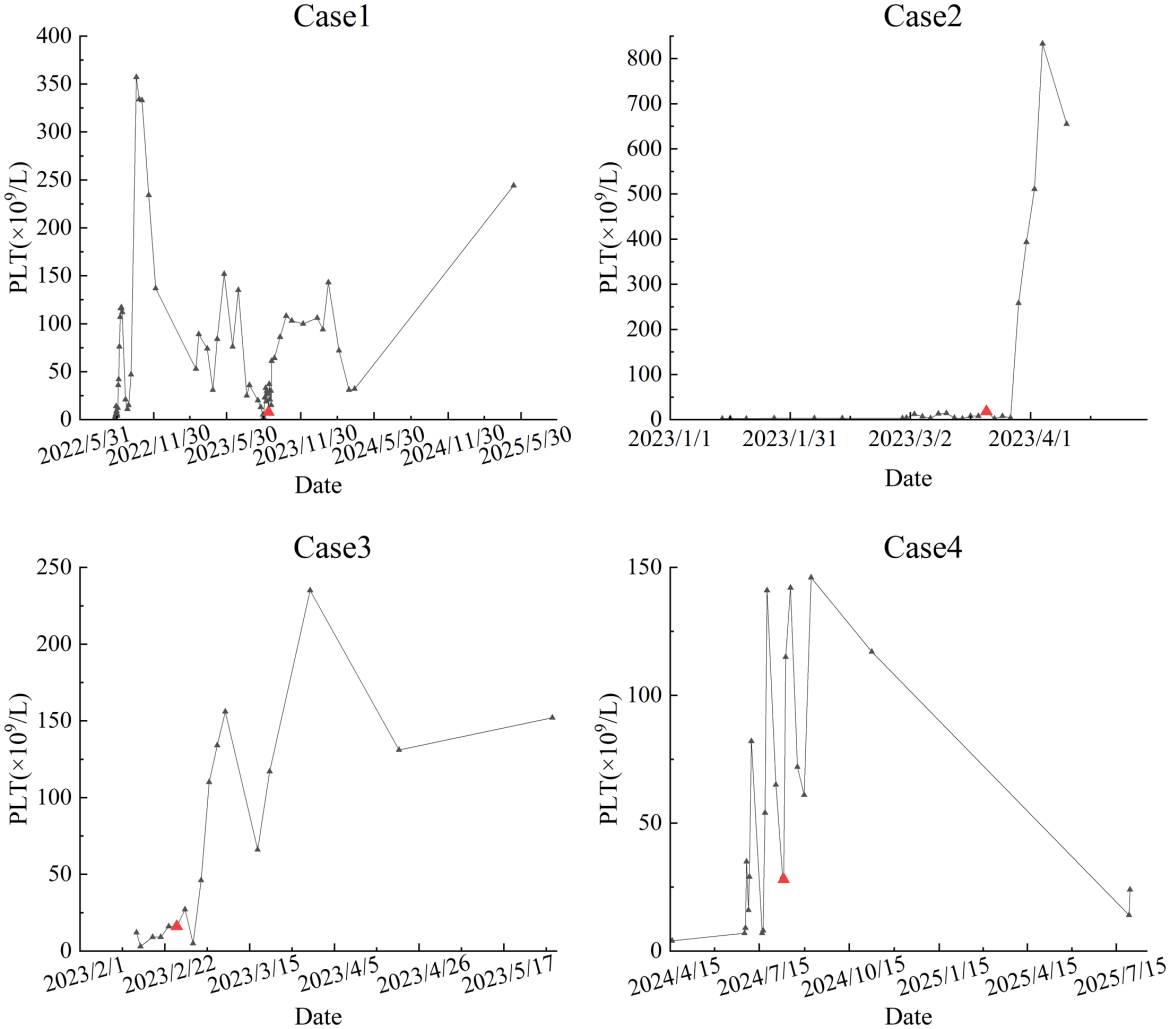
Platelet (PLT) numbers after anti-CD20 monoclonal antibody combined with hetrombopag treatment. The red triangle indicates the start of combination therapy.

Figure 1 shows the flow diagram of treatment. Figure 2 shows the PLT counts after treatment with anti-CD20 monoclonal antibody and hetrombopag.

## 4 Discussion

We retrospectively included four patients with ITP; the median disease duration is 9 months. Their prior lines of therapy were numbered 4, 2, 4, and 2, respectively. In this report, all four patients achieved CR, with the TTR ranging from 2 to 9 days, the DoR ranging from 3 to 27 months, and a good safety profile. Our findings indicate that combination therapy with anti-CD20 monoclonal antibody and hetrombopag may provide therapeutic benefits in patients with ITP. Although both anti-CD20 monoclonal antibody and TPO-RA are second-line agents, they have different time windows of action. Specifically, anti-CD20 monoclonal antibodies have a longer DoR and can produce a sustained response, while TPO-RA has a rapid onset of action but questionable long-term effectiveness. Relapsed/refractory ITP may benefit from combination therapy with anti-CD20 monoclonal antibody and TPO-RA (22–25). Notably, ITP involves two distinct pathophysiological mechanisms: immune-mediated PLT destruction and insufficient PLT production (1). Given that anti-CD20 monoclonal antibody and TPO-RA target these two pathophysiological mechanisms, their combination may yield enhanced efficacy. Anti-CD20 monoclonal antibodies may allow long-term remission by decreasing PLT destruction through immunomodulatory effects, depleting B cells, increasing CD4^+^ regulatory T cells, and downregulating dendritic cells. However, it may take several weeks to achieve an increase in PLT count. Contrastingly, TPO-RA primarily stimulates PLT production and promotes a rapid increase in PLT counts, which is usually not sustained in most patients after TPO-RA discontinuation. Accordingly, the rapid increase in PLT count can safely override the wait for a response to other treatments (e.g., anti-CD20 monoclonal antibody), or, if feasible, splenectomy can be safely performed. Taken together, combination therapy with anti-CD20 monoclonal antibody and TPO-RA may have synergistic effects on maintaining normal PLT levels. Similarly, combination therapy with low-dose rituximab and rhTPO has a shorter onset of action than rituximab therapy alone; however, there was no significant between-protocol difference in the long-term response rates (26). Taken together, combination therapy with anti-CD20 monoclonal antibody and TPO-RA may provide a novel treatment for patients with relapsed/refractory ITP.

Yanmei et al. (27) reported that combination therapy with eltrombopag and rituximab yielded a higher achievement rate of R than eltrombopag alone (72.3% vs. 54.0%, *p* = 0.042); furthermore, the OR was also higher in the combination therapy group after 12 weeks of treatment (80.0% vs. 64.0%, *p* = 0.055). Moreover, Kikuchi et al. reported that, in Japan, the addition of rituximab to standard treatment with romiplostim and splenectomy reduced treatment costs and improved outcomes in adults with ITP compared with standard treatment alone (28). Another study of patients with refractory primary ITP demonstrated that a combination of TPO-RA with rituximab showed favorable efficacy and prognosis (29). Among 11 patients receiving combined treatment with romiplostim and rituximab, 9 patients achieved long-term remission without requiring romiplostim maintenance therapy, while 1 patient refused continued treatment owing to severe complications (22–24, 30, 31).

We included four patients; among them, three and one had primary and secondary ITP, respectively. Notably, all four patients achieved CR with a TTR of 2, 9, 9, and 2 days, respectively. The four cases received the same anti-CD20 monoclonal antibody dose, of which two received rituximab and the other two received ripertamab. This study could not demonstrate a relationship between TTR variability and anti-CD20 monoclonal antibody type. The times to CR was 44, 9, 9, and 2 days, respectively, while the corresponding DoRs were 23, 27, 29, and 12 months. Our findings indicated that combination therapy with anti-CD20 monoclonal antibody and hetrombopag has relatively good efficacy in patients with ITP. Case 3 presents with ITP secondary to Sjögren’s syndrome, making immunosuppressive therapy necessary. The significant platelet increase observed within a short treatment window suggests a greater likelihood of efficacy from the combination of anti-CD20 monoclonal antibody and hetrombopag. However, her response could not completely rule out the influence of immunosuppression; therefore, further prospective studies are required for definitive confirmation. During combination therapy, PLT levels usually decreased after the initial increase; however, this was mostly temporary. After the completion of combination therapy, patients can use hetrombopag alone at a small dose to maintain normal PLT levels, and selected patients may achieve sustained remission. Among the four patients in this study, cases 1 and 2 experienced relapse following the completion of anti-CD20 monoclonal antibody and hetrombopag combination therapy. After anti-CD20 monoclonal antibody-mediated depletion of peripheral B cells, some patients exhibited rapid B-cell reconstitution, restoring their capacity to produce antiplatelet antibodies and leading to disease relapse. Case 1 relapsed after 6 months, demonstrating relatively short-term sustained efficacy, which may be attributed to B-cell reconstitution and functional recovery.

Currently, there are two commonly used dosing regimens for anti-CD20 monoclonal antibody in ITP treatment. The first is a standard-dose regimen involving four weekly intravenous (IV) doses at 375 mg/m^2^, which usually exerts effects 4–8 weeks after the initial dose. The second is a low-dose regimen that involves 100 mg IV once a week for four doses or 375 mg/m^2^ IV once a week with a slightly longer onset of action. Compared with standard doses, low-dose rituximab significantly improves coagulation indicators and is less toxic and safer (11). In our study, the anti-CD20 monoclonal antibody was administered at a pulse dose of 375 mg/m^2^ in the first week and 100 mg/week for the next 3 weeks, which ensured efficacy and minimized the risk of adverse reactions.

This study has some limitations. First, this was a case series (*n* = 4) that cannot be used to infer comparative efficacy. Other limitations include its small sample size, heterogeneity of prior/concomitant therapies (e.g., tacrolimus/hydroxychloroquine in case 3), and the absence of a control group. A large-scale randomized controlled trial is warranted to validate the safety and efficacy of this combination regimen.

## 5 Conclusion

Our findings showed that a combination therapy involving anti-CD20 monoclonal antibody and hetrombopag was effective in patients with ITP and was well-tolerated. However, a large-scale prospective study is warranted to better evaluate the efficacy and safety of this treatment.

## Data Availability

The raw data supporting the conclusions of this article will be made available by the authors, without undue reservation.
